# Idiopathic Bilateral Adrenal Hemorrhage in a 63-Year-Old Male: A Case Report and Review of the Literature

**DOI:** 10.1155/2015/503638

**Published:** 2015-04-21

**Authors:** Naveen Dhawan, Vijay Kumar Bodukam, Kshitij Thakur, Amandeep Singh, Donald Jenkins, Jaya Bahl

**Affiliations:** ^1^Nova Southeastern University Health Sciences Division, Fort Lauderdale, FL 33314-7796, USA; ^2^Crozer-Chester Medical Center, Upland, PA 19013, USA; ^3^Florida International University (FIU), North Miami Beach, FL 33199, USA

## Abstract

Adrenal hemorrhage is a largely uncommon condition typically caused by a number of factors including infection, MI, CHF, anticoagulants, trauma, surgery, and antiphospholipid syndrome. Yet, idiopathic bilateral hemorrhage is rare. The authors present a case of a 63-year-old male who presented with abdominal pain that was eventually diagnosed as bilateral adrenal hemorrhages due to an unknown origin. Abdominal CT revealed normal adrenal glands without enlargement, but an MRI displayed enlargement due to hemorrhage in both adrenals. There was no known cause; the patient had not suffered from an acute infection and was not on anticoagulants, and the patient's history did not reveal any of the other known causative factors. The case underscores the importance of keeping bilateral adrenal hemorrhages on the list of differentials even when a cause is not immediately clear. It also raises the question of whether CT is the most sensitive test in the diagnosis of adrenal hemorrhage and whether the diagnostic approach should place greater weight on MRI. The case highlights the need for prompt therapy with steroids once bilateral hemorrhage is suspected to avert the development or progression of adrenal insufficiency.

## 1. Background

Adrenal hemorrhage is a serious condition that can result in adrenal insufficiency, shock, acute adrenal crisis, and mortality if not managed with adequate treatment [1, 2]. Patients often present with nonspecific signs and symptoms and the diagnosis can only be confirmed during autopsy [3]. Adrenal hemorrhage may be caused by several factors including infection, MI, CHF, anticoagulants, trauma, surgery, and antiphospholipid syndrome [4]. Few case reports have presented bilateral adrenal hemorrhage due to an unknown cause. Since the approach to management is often guided by the cause, this presents with a therapeutic dilemma. We describe a case of bilateral adrenal hemorrhage that was an incidental finding on MRI for further evaluation of intractable abdominal pain and could not be detected using CT. The patient was not on anticoagulants and had not suffered from any acute infection. His history did not show any of the other known causative factors. Thus, no known cause of hemorrhaging was determined.

## 2. Case Presentation

A 63-year-old Caucasian male presented to our hospital complaining of band-like abdominal pain. He reported that earlier in the day he felt normal but developed a sudden midabdominal pain around the umbilical area bilaterally. The pain was described as being crampy to sharp, 10/10 in severity, and nonradiating, with no aggravating factors, but alleviated slightly by pain medications in the ED. He reported constant pain with nausea and vomiting which also is associated with profuse sweating. The patient was feeling fine the days prior to admission. The review of systems was otherwise unremarkable. His past medical history included type II diabetes mellitus and duodenal ulcer at the age of 21. Current medications included metformin.

On physical examination, the temperature was 97.5°F, blood pressure was 169/76 mmHg, and the pulse was 84 beats per minute. The patient was alert and oriented to time, place, and person. The abdomen was soft with normal bowel sounds. He showed tenderness to deep palpation in the epigastric and bilateral midabdomen and periumbilical area.

On BMP examination, the potassium was low at 3.3, glucose was elevated at 387, and BUN was elevated at 21. Additionally lipase was 65 and magnesium was 1.8. The first set of troponin was not detected. The pro-BNP was 492 while the lactic acid was elevated at 2.8. The UA revealed glucose >500 and protein level at 100. Random cortisol level upon admission was 19.1 and 4.6 (low) on day 1 after admission. The labs revealed an elevated WBC at 14.7. The PT/INR was normal. Over the period of the patient's care, the hemoglobin levels decreased to 13.3 g/dL, while the platelet count also decreased to 107. An abdominal flat plate series did not show any obvious air-fluid levels or any obstruction. A lupus anticoagulant test was negative. The patient was not tested for antiphospholipid antibodies as antiphospholipid syndrome (APS).

**Figure 1:**
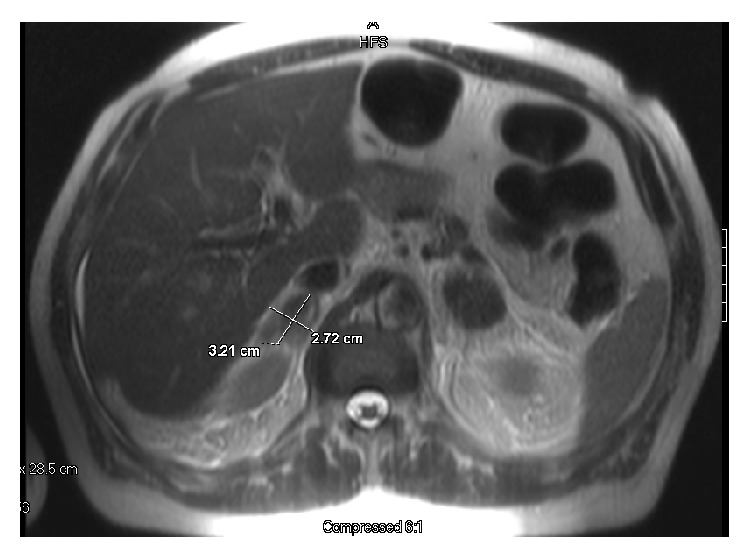
Enlargement of adrenal gland with possible adrenal hemorrhage.

Computed tomography (CT) with contrast of the abdomen and pelvis was conducted which showed nonspecific mild infiltration of fat within the retroperitoneum inferior to the pancreas. It was suspected that this likely represented a mild-acute inflammatory process. Thus, the initial differential diagnosis was pancreatitis and correlation with pancreatic enzymes was advised. An MRI was later conducted which revealed enlargement of both adrenal glands most likely from bilateral adrenal hemorrhage. Secondary causes included gallstone and widely patent celiac, superior mesenteric, and inferior mesenteric arteries, but these were ruled out. The right adrenal measured approximately 3.2 × 2.7 × 4.5 cm. The left adrenal measured 3.3 × 3.9 × 4.8 cm. Neither adrenal enhanced following contrast administration, Figure 1.

Importantly, the patient did not show signs of adrenal insufficiency. Yet, given the enlargement of the adrenals, the patient was treated with hydrocortisone (20 mg in morning, 10 mg in evening) that substituted for the loss of both corticosteroid and mineralocorticoid activity of the adrenal glands after the expert consultation by endocrinologist. Close outpatient follow-up was recommended with an endocrinology for continued management.

## 3. Discussion

Adrenal hemorrhage is an uncommon condition that is of serious concern as it may result in adrenal insufficiency and death. Adrenal hemorrhage may be caused by several factors including infection, MI, CHF, anticoagulants, trauma, surgery, and antiphospholipid syndrome [4]. The differential for adrenal hemorrhage includes granulomatous infection, amyloidosis, septic shock, MI, deposits from metastatic tumor, infiltrative diseases (i.e., lymphoma and leukemia), adrenal crisis, pheochromocytoma, adrenal adenoma, and adrenal carcinoma [5]. Heparin-induced thrombocytopenia (HIT) has also been reported as a cause of bilateral adrenal hemorrhage. Ketha et al. [6] recently reported a case of 4 patients that developed bilateral adrenal hemorrhage one week after surgery during which postoperative heparin was not administered, indicating spontaneous heparin-induced thrombocytopenia (HIT) as a likely cause.

The underlying disease mechanism in cases of adrenal hemorrhage has not been fully elucidated. The term “adrenal dam” has been ascribed to vascularities within the adrenals, implying the large degree of adrenal vascularities that are susceptible to hemorrhage due to their distinct anatomical attributes [7, 8]. While some have suggested that reduced capillary resistance as a result of aging may be a factor [9], others have postulated that elevated catecholamines and ACTH as a result of stress produce vasoconstriction and platelet aggregation and lead to reperfusion and subsequent bleeding, particularly in the capillaries within the distal corticomedullary junction [10–12].

Several signs are prominent during the initial stages of adrenal hemorrhage. Patients may present with fever, nausea, vomiting, weakness, dizziness, tachycardia, anorexia, fatigue, back pain, and epigastric pain [7, 10]. Most cases may not display adrenal insufficiency upon diagnosis [7]. Yet, in order to determine adrenal insufficiency, a random cortisol test can be performed; a serum cortisol level >34 *μ*g/dL would exclude the condition, while a serum cortisol level <15 *μ*g/dL may be confirmatory [13]. Hypotension, hyponatremia, hypoglycemia, and hyperkalemia may be important indicators of adrenal insufficiency [10, 14].

Importantly, our case raises the question of how abdominal pain relates to adrenal hemorrhage of an idiopathic nature. Our patient presented with band-like abdominal pain as a presenting symptom. In the case of a 46-year-old male with bilateral adrenal hemorrhage described by Dahiya et al. [15], the patient also presented with abdominal pain. However, it is important to note that the patient described by Dahiya et al. had a history of GERD. One reliable sign of adrenal hemorrhage has been pyrexia [8]. Thus, further reexamination of the range of nonspecific signs and symptoms in adrenal hemorrhage cases may be warranted.

The standard diagnostic assessment includes computed tomography (CT) with or without the use of IV contrast, which would display round enlargements of the adrenals with varying densities [16, 17]. The threshold for adrenal mass is 4 cm, which has a low specificity but a sensitivity of 90% [16]. Several papers have supported magnetic resonance imaging (MRI) as a test with more accuracy for diagnosing adrenal hemorrhage, as it can even differentiate chronic from subacute hemorrhages [4, 16, 18, 19]. Our experience is similar to that of Dahiya et al. [15], who reported a case of spontaneous bilateral adrenal hemorrhage in a 46-year-old male in which CT (both with and without contrast) failed to display hemorrhage as the cause of enlargement. Thus, the question of CT as a definitive modality in identifying adrenal hemorrhages remains pertinent.

The treatment of adrenal insufficiency due to adrenal hemorrhage is typically hydrocortisone given through parenteral administration [20]. Mineralocorticoids may be added, but prolonged administration of mineralocorticoids is typically not needed in patients that have developed adrenal insufficiency when caused by bilateral hemorrhage [21]. Although our patient did not display adrenal insufficiency, we promptly administered hydrocortisone to help bolster adrenal function in the event of impeding progression to adrenal insufficiency.

Relatively few cases of idiopathic bilateral adrenal hemorrhage have been reported in the worldwide literature [5, 7, 15, 17, 22]. Ogino et al. [7] previously described a 54-year-old Japanese woman who was diagnosed with bilateral adrenal hemorrhage due to an unknown cause that resolved 6–12 months later following hydrocortisone therapy. A previous case described an 80 year-old woman with bilateral massive adrenal hemorrhage previously described a patient who presented with constitutional symptoms and was successfully treated with steroid replacement therapy [5]. Dahiya et al. [15] recently described the case of a 46-year-old with spontaneous idiopathic bilateral adrenal hemorrhage which resulted in fatality due to lack of early recognition. Previous reports have also described idiopathic spontaneous adrenal hemorrhage (SAH) during the last trimester in pregnancy [22]. SAH typically occurs without any injury or tumor in the adrenal glands. SAH usually involves the right adrenal gland with a reported incidence of 0.14–1.1% in pregnancies [22, 23].

## 4. Conclusion

In summary, we have presented the case of a 63-year-old man with bilateral adrenal hemorrhage due to an idiopathic cause. Hydrocortisone therapy was employed to account for the lack of adrenal function even though at the time the patient did not show adrenal insufficiency. Thus, the case illustrates the importance of prompt therapy upon diagnosis. While CT was conducted, it did not show a significant finding. An MRI ultimately revealed the diagnosis. This case illustrates the importance of proper imaging and raises the question of appropriate imaging modality for the diagnosis of adrenal hemorrhage. It also underscores the importance of acute management of suspected bilateral adrenal hemorrhage with hydrocortisone even in the absence of adrenal insufficiency.
